# Sexual and Reproductive Health for Women Who Live With Inflammatory Bowel Disease: An Integrative Review

**DOI:** 10.1002/nop2.70269

**Published:** 2025-06-27

**Authors:** Kate O'Reilly, Susan McInnes, Eleanor Holroyd, Kath Peters

**Affiliations:** ^1^ Western Sydney University Penrith Australia; ^2^ Auckland University of Technology Auckland New Zealand

**Keywords:** inflammatory bowel disease, sexual and reproductive health, women

## Abstract

**Aims:**

To provide an understanding of the sexual and reproductive health issues for women who live with inflammatory bowel disease.

**Design:**

Whittemore and Knaffle's integrative review.

**Methods:**

Databases searched in this integrative review included: CINAHL Plus, Google Scholar, SCOPUS and Web of Science databases. A defined research aim guided the search strategy and facilitated the accurate extraction of data from primary qualitative and quantitative research conducted between 2012 and 2022.

**Results:**

Nineteen papers were included in this review. All papers were appraised using the Mixed Methods Appraisal Tool and were found to be of good methodological quality. The following three themes were identified: (1) inflammatory bowel disease negatively impacts sexual, reproductive and social health; (2) inflammatory bowel disease and sexual functioning and (3) reproductive knowledge and reservedness to discuss sexual health. This review of the literature highlights that sexual and reproductive health issues for women who live with inflammatory bowel disease are very focused on sexual dysfunction and reproduction. The review illuminates that there is a dearth of information that explores women's sexuality more broadly across the lifespan.

## 1 Introduction

Inflammatory bowel diseases (IBD) including Crohn's disease (CD) and ulcerative colitis (UC) are chronic gastrointestinal diseases which present as unpredictable relapsing conditions (Woods 2022). While CD and UC are discreet diseases, they both include inflammation of the intestinal tract which is characterised by several symptoms including, but not limited to, abdominal pain, rectal bleeding, diarrhoea, constipation, bloating and fatigue, all of which significantly impact on quality of life (Woods 2022; Department of Health 2017). The onset of IBD typically occurs during adolescence to early adulthood (Department of Health 2017). However, there are significant variations to this with the Global Disease Burden 2017 Inflammatory Bowel Disease Collaborators (2020) highlighting that onset can span as many as four decades. The cause of IBD is not fully known although the interaction between genetic, environmental, immunological and infectious factors in susceptible people are identified by researchers to be the likely causes (Department of Health 2017; Global Disease Burden 2017 Inflammatory Bowel Disease Collaborators 2020). Globally, it is estimated that the prevalence of IBD is 6.8 million (Global Disease Burden 2017 Inflammatory Bowel Disease Collaborators 2020). The prevalence of IBD worldwide is expected to continue rising due to a lower mortality rate and increasing treatment options. In Australia in 2022, the prevalence of IBD was estimated to be 100,000 persons (Department of Health 2017). In New Zealand in 2017 it was estimated there were 20,792 people living with IBD. This equates to 1 in 227 people with estimates of a 5.6% increase in cases each year (Kahui et al. 2017). In a study which included 195 countries and territories, the prevalence of IBD in all years from 1990 to 2017 is higher in females (57%) compared to males (43%) (Global Disease Burden 2017 Inflammatory Bowel Disease Collaborators 2020). DiGiacomo et al. (2015) highlight that health systems have been operationalised to respond to acute care needs rather than chronic conditions. Furthermore, health services have been critiqued as failing to offer a gender‐based approach which may negatively impact on health outcomes with women at a higher risk of ill health than men (Australian Government 2023; Merone et al. 2022).

While it is recognised that IBD can have far reaching negative impacts on a person's life such as anaemia, debilitating fatigue, pain and bowel obstruction regardless of sex (Department of Health 2017), it is recognised within the literature that sociocultural factors must also be considered in providing comprehensive person‐centred health care (DiGiacomo et al. 2015). Women have specific sexual and reproductive needs that differ across their lifespans, yet sexual, and reproductive health for women who live with IBD remains a topic which is underexplored. The World Health Organisation (WHO) (2015, 5) define sexual health or sexual wellbeing as ‘physical, emotional, mental and social wellbeing in relation to sexuality; it is not merely the absence of disease, dysfunction or infirmity’. Reproductive health is also defined in the same way with the addition that it encompasses ‘all matters relating to the reproductive system and to its functions and processes’ (United Nations 1995, 40).

## 2 Aim

The aim of this paper is to review the available literature regarding the sexual and reproductive health experiences and perceptions of women who live with IBD. The broad and inclusive definitions of sexual and reproductive health offered by the WHO (World Health Organisation 2015; United Nations 1995) have informed the review.

## 3 Methods

The integrative review framework described by Whittemore and Knafl (2005) was used to guide the conduct of the literature search; no protocol was registered for this review. Integrative reviews are appropriate when the simultaneous inclusion of experimental and non‐experimental studies is warranted to capture a complete understanding of the phenomenon under examination (Whittemore and Knafl 2005). A defined research aim guided the search strategy and facilitated the accurate extraction of data from primary research papers.

### 3.1 Data Collection

CINAHL Plus, Google Scholar, SCOPUS and Web of Science databases were used to identify primary research projects that explored sexual and reproductive health in women diagnosed with inflammatory bowel disease. Search terms included women's health or reproductive health or female health or sexual health AND inflammatory bowel disease OR ulcerative colitis OR Crohn's disease AND experiences or perceptions or attitudes or views. These broad terms were chosen to align with definitions of sexual and reproductive health from the WHO (World Health Organisation 2015; United Nations 1995). To capture contemporary research, the search was limited to primary research reported in English and conducted between January 2012 and December 2023 as presented in Table 1.

**TABLE 1:** Inclusion/exclusion criteria.

Inclusion criteria	Exclusion criteria
10 Years (2012–2023)	Reviews/editorials/book chapters/discussion papers
English	Only reports on male subjects
Primary research	Could not extrapolate female from male data
Peer reviewed	Focus on a particular condition (e.g., HIV, cancer, arthritis)
Full text available	Focus on stomas
	Focus on preconception/pregnancy

**FIGURE 1:**
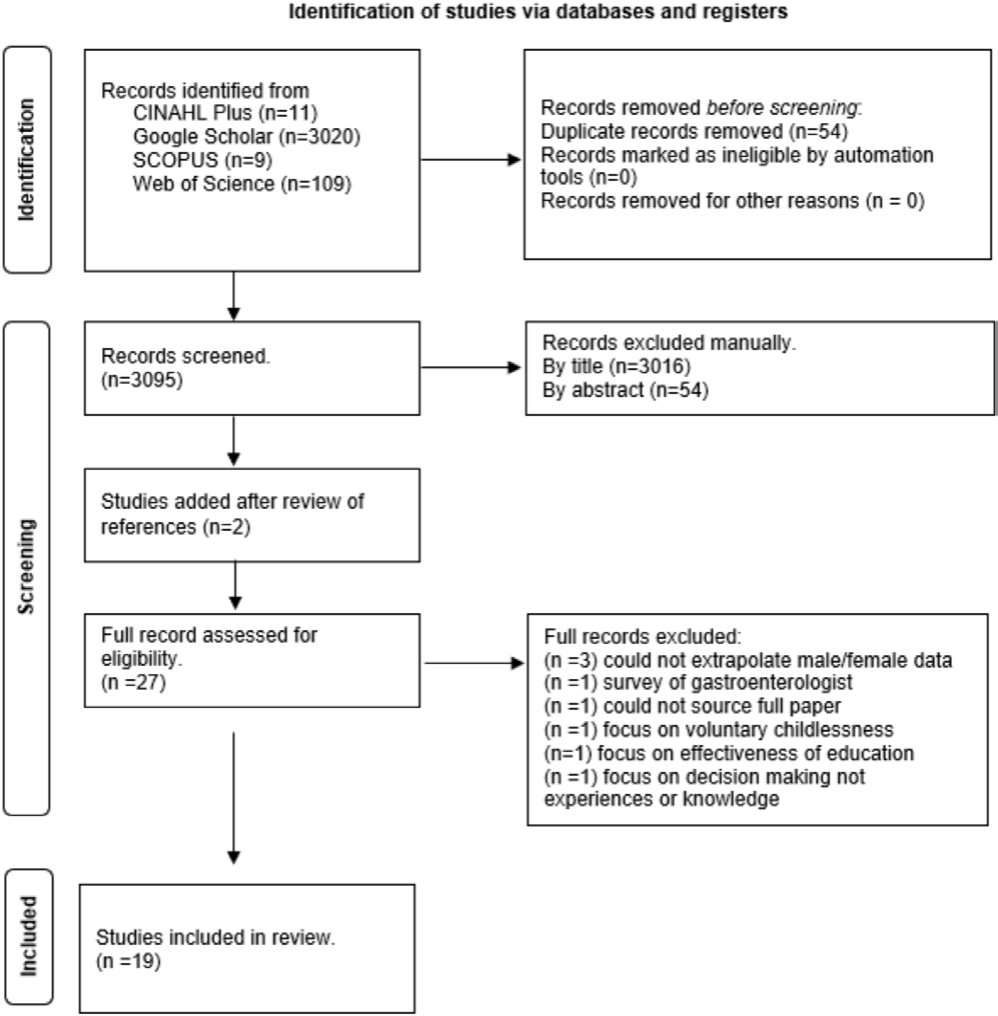
Diagrammatic representation of search strategy and screening.

Figure 1 presents the Preferred Reporting Items for Systematic Reviews and Meta‐Analyses (PRISMA) flow diagram (Page et al. 2021). PRISMA ensures transparency in the review of studies irrespective of the design of included studies (Page et al. 2021). For the integrative review, the PRISMA checklist for systematic reviews was used and adhered to as closely as possible (see Supporting Information). In total, the search identified 3149 papers. Duplicates were removed (*n* = 54) leaving a total of 3095 papers which were screened by title. The manual screening by title resulted in the removal of 3016 papers, leaving 79 papers. These 79 papers were then screened by abstract, which resulted in the removal of an additional 54 papers, leaving 25 papers. Two papers were added for potential inclusion after the review of reference lists (*n* = 27). Twenty‐seven papers were read in full to assess for eligibility, with eight papers removed as they did not meet the inclusion criteria. This left a total of 19 papers which were included in this review (see Table 2).

**TABLE 2:** Summary of papers included in the literature review.

References	Country	Aim	Sample	Method	Findings
IBD negatively impacts sexual, reproductive and social health					
Calvet et al. (2018)	Spain	To assess patients' perceptions of the impact of UC on social and professional lives	585 patients	Quantitative cross‐sectional survey	Participants completed the UC‐LIFE survey High proportions of patients considered their disease ‘sometimes’, ‘frequently’ or ‘mostly/always’ influenced leisure activities (65.1%), recreational or professional activities (57.6%), or relationships with relatives or friends (9.9%). 7.4% of men and 28.4% of women reported that UC had influenced their decision to have children. The percentages of patients reporting a negative impact of UC were statistically significantly greater in women than in men (*p* < 0.001)
Knowles et al. (2013)	Australia	To characterise the relationships between illness perceptions, body image and self‐consciousness, sexual health (sexual problems and sexual satisfaction), anxiety and depression, and marital and family functioning in patients with IBD	61 females 13 males	Quantitative online survey	The Sexual Problems Scale (SPS) was used to assess sexual arousal and orgasm. Body Image and Self‐Consciousness During Intimacy Scale (BISC) assessed levels of concern associated with body image during sex. Females reported significantly more sexual problems than males [*F*(1,72) = 39.15, *p* < 0.001]. 7 males (53.9%) and 51 females (83.6%) identified a lack of sexual interest. 34 females (55.7%) identified experiencing difficulty having an orgasm Unexpectedly, the direct influence of gender suggested that being female was associated with greater sexual problems
Roseira et al. (2020)	Portugal	To assess sexual quality of life (SQoL) in IBD patients compared with healthy controls	458 females 389 males 399 healthy controls (HC)	Quantitative cross‐sectional study	SQoL was assessed using the Sexual Quality of Life questionnaire Male/Female (SQoL M/F) Inflammatory bowel disease patients reported lower SQoL (men: 77.29 vs. 83.83; *p* < 0.001; women: 70.40 vs. 81.63; *p* < 0.001) compared with controls. Women with IBD reported being satisfied with the frequency of sexual activity (78%). In women only, depression was significantly associated with SQoL (*β*, −1.97; 95% CI 2.31–1.63; *p* < 0.001).
IBD and sexual functioning					
Bel et al. (2015)	Netherlands	To evaluate sexual function and its association with depression among patients with IBD	168 females 119 males	Quantitative web‐based survey	Sexual function in women was assessed with the Female Sexual Function Index (FSFI) Female patients with active disease reported more lubrication problems and more dyspareunia compared with patients in remission and controls. In the total group of women with IBD, 51.2% had sexual dysfunction, against 44.3% of the control group, *χ* ^2^ (1) = 1.22, *p* > 0.05 Of the women with active IBD, 63.1% had sexual dysfunction (total score < 26.55), against 44% of women in remission, *χ* ^2^ (1) = 5.74, *p* < 0.05. Patients with more active disease reported more sexual problems Patients reporting higher quality of life reported less sexual problems. Patients scoring higher on fatigue, depression and negative body image reported more sexual problems
Boudiaf et al. (2021)	France	To compare the prevalence of sexual dysfunction (SD) in Crohn's disease patients with active perianal disease (PD) versus controls without active PD	64 women, 33 men 238 controls	Quantitative survey‐based cross‐sectional study	Women completed the Female Sexual Function Index (FSFI). SD was found in 66% of the female patients versus 50% of the controls (*p* = 0.04). The predictive factor most strongly associated with SD in women was severely active perianal disease (PD). Pain, soiling and faecal incontinence were the main complaints in both men and women. Only 27% of women had discussed sexual problems with their gastroenterologist or proctologist More than half of the women with PD wanted information about the impact of PD CD on their intimacy and sexual health (56%)
Bulut and Törüner (2019)	Turkey	To show the effect of disease type and activity on sexual life and QoL in patients with IBD	122 patients with IBS 42 control	Quantitative cross‐sectional study	All participants completed the Arizona Sexual Experience Scale (ASEX). The mean ASEX scores of women with UC (19.59 ± 7.59) and CD (19.38 ± 6.27) were both significantly higher than those of the control group (15 ± 4.76). Women in the control group had better sexual desire, arousal, vaginal lubrication and orgasm than those in the UC and CD groups. The ASEX mean score was higher in women with active disease
Domislovic et al. (2021)	Croatia	To assess the prevalence of sexual dysfunction (SD) and erectile dysfunction (ED), QOL, their predictors and age‐related dynamic in IBD patients	122 male patients 80 female patients	Quantitative cross‐sectional study	Female sexual functioning was assessed using Female Sexual Functioning Index (FSFI). QOL was assessed using IBDQ‐32 through bowel, systemic, emotional and social domains. Female patients with SD had significantly lower total QOL score and systemic, emotional and social QOL score Prevalence of SD was considerably high in women (75.0%). No difference in SD or ED rates between CD and UC were observed
Eluri et al. (2018)	United States	To evaluate patient‐reported interest in sexual activity and satisfaction with sex life in a large cohort of IBD patients	3811 individuals	Quantitative cross‐sectional study	Sexual Function and Satisfaction (SexFS) scale was used. 2581 (68%) individuals completed the survey. The mean PROMIS score for interest in sexual activity was 41 for women with CD 40 for women with UC, comparable to the US general population mean of 42 Satisfaction with sex life was lower for women with IBD (mean *T* scores of 47 for CD and 46 for UC) compared to the population mean of 49, *p* < 0.01. Factors associated with lower sexual interest scores in patients with UC and CD were female sex, increasing age and fatigue (*p* < 0.05 for all). Increasing anxiety was also found to be significantly associated with lower sexual interest scores in patients with UC (*p* = 0.03). Sexual interest and satisfaction scores were positively associated with disease‐specific quality of life
Marín et al. (2013)	Spain	To evaluate the prevalence and predisposing factors of sexual dysfunction among IBD patients, and their own perception	202 females 153 males 200 controls	Quantitative cross‐sectional study	Sexual function was assessed using the Female Sexual Function Index (FSFI) in women 39% of the women and 35% of the men who were sexually active at the time of IBD diagnosis acknowledged that the disease changed (worsened) their sexual life A greater proportion of women than men felt that sexual desire had decreased after IBD diagnosis (47 vs. 29%, *p* = 0.001) 46% of the women and 30% of the men declared that their sexual satisfaction worsened after disease diagnosis (*p* = 0.044) Among those patients who felt that intimacy had worsened because of IBD, fatigue was the main complaint in both men and women Women attributed worsening intimacy female disease‐related symptoms (i.e., abdominal pain, diarrhoea, or incontinence). 61% percent of the women and 46% of the men declared that information about the impact of IBD on intimacy and sexuality should be given at IBD diagnosis. 49% of the patients with IBD presented an abnormal FSFI score as defined by a total score lower than 26 points, versus only 19% of the controls (*p* = 0.0001)
Nisihara et al. (2020)	Brazil	To study the prevalence of sexual disorders in a sample of Brazilian male and female patients with IDB and its association with depression	40 females and 40 males with IBD 112 controls	Quantitative cross‐sectional study	Female Sexual Function Index (FSFI) assessed sexual function. Beck Depression Inventory‐II was used to access depression. 82.5% of females had sexual dysfunction versus 45.8% in healthy controls (*p* = 0.0002; OR = 5.5; 95% CI 2.1–14.2). When the number of males with IBD with any degree of sexual dysfunction (70%) were compared with the number of females with IBD with any degree of sexual dysfunction (82.5%) no differences were found. Among females, 20% had mild, 17.5% moderate and 15% had severe depression
Nøhr et al. (2020)	Denmark	To investigate the risk of sexual dysfunction in women with IBD	38,011 females	Quantitative cross‐sectional study	Sexual health questions were adapted from the Danish National Health Survey. Of the study population (38,011 women) 196 (0.5%) had CD and 409 (1.1%) had UC. Compared to women without IBD, women with UC did not have significantly decreased sexual function Women with CD had more difficulty achieving orgasm (adjusted odds ratio [aOR] 1.53; 95% confidence interval [CI] 1.02–2.30), increased dyspareunia (aOR 1.71; 95% CI 1.11–2.63) and deep dyspareunia (aOR 2.00; 95% CI 1.24–3.22)
Riviere et al. (2017)	France	To compare rates of sexual dysfunction (SD) between IBD patients and healthy controls (HC)	192 females 166 males 110 HC	Quantitative cross‐sectional study	Female Sexual Function Index (FSFI) assessed sexual function in participants In women, SD was identified in 103/192 (53.6%) IBD patients, 15/53 (28.3%) of HC (*p* < 0.01) and 38/49 (77.6%) IBS patients (*p* = 0.1 vs. IBD) Predictors of SD were social and emotional functioning and anxiety in women. Female patients with IBD scored significantly lower than HC in sexual desire, arousal and orgasm (*p* < 0.01, *p* = 0.01 and *p* = 0.02, respectively). Lubrication and dyspareunia scores in IBD patients were not significantly different from those of HC (*p* = 0.17 and *p* = 0.32, respectively). In women, no difference of SD rate was observed between patients with active or inactive disease (53.2% vs. 61.2%, *p* = 0.66). 28.4% of women and 15.2% of men considered that the impact of their disease on their sexual function was negative
Shmidt et al. (2019)	United States	To describe sexual function at baseline and over time in a prospective inception cohort of adult women with IBD	116 females	Quantitative cross‐sectional study	Female Sexual Function Index (FSFI) was used. Ninety‐seven percent of women had sexual dysfunction. Prevalence of sexual dysfunction at baseline was similar in CD and UC (58 [97%] CD patients, 54 [96.4%] UC patients; *p* = 1.00) and remained unchanged throughout the 2‐year duration of the study (*p* = 0.99). Abdominal pain or diarrhoea over the past 4 weeks were associated with changes in sexual function (*p* = 0.24, *p* = 0.40, respectively, in all women)
Reproductive knowledge and reservedness to discuss sexual health					
Ellul et al. (2019)	Malta, Greece, Israel, Italy, Portugal and Spain	To assess the perspectives of IBD patients on fertility, pregnancy and its outcomes	384 female IBD patients	Quantitative prospective, cross‐sectional study	Participants completed a survey based on European Crohn's and Colitis Organisation (ECCO) guidelines on pregnancy. The questionnaire consisted of eight sections related to fertility, consideration of pregnancy, pregnancy and its outcomes, delivery, breastfeeding, surgery, contraception and cervical pathologies. 50% of participants had a diagnosis of ulcerative colitis, 49.4% had CD and 0.6% patients had a diagnosis of indeterminate colitis. 63.1% of participants were unsure if medication should be stopped during pregnancy. Only 9.9% of patients answered that treatment should not be stopped during pregnancy. Voluntary childlessness included personal views and serious fears, such as IBD and/or IBD medications causing harm to the baby, passing on IBD to the baby, having a complicated pregnancy because of IBD, and/or not being able to take care of the baby because of IBD. The breastfeeding rate among IBD patients was 29.62%. In the cohort, 145 patients were counselled about the use of contraception and only 39 (27%) patients used a contraception method. Only 17% of patients were counselled on the benefits of undergoing regular Pap tests
Huang et al. (2015)	Canada	To examine the effects of IBD‐specific reproductive knowledge and discussion of family planning with a physician on childlessness among women with IBD	248 females	Quantitative cross‐sectional survey	Participants completed the Crohn's and Colitis Pregnancy Knowledge questionnaire (CCPKnow). CD was present in 60.5% (150 of 248), UC in 35.0% (88 of 248) and indeterminate colitis in 4.0% (10 of 248) of the respondents. Slightly more than one‐half (128 of 248 [51.6%]) of the women were childless The prevalence of childlessness was higher among patients who were diagnosed with IBD before 18 years of age (*p* < 0.001), had a history of ostomy (*p* < 0.016) or colectomy (*p* = 0.034), and among those working full‐time (*p* = 0.02), those with poor CCPKnow scores (*p* = 0.008) and those without partners (*p* = 0.001). 131 of 248 (52.8%) had poor CCPKnow scores. The prevalence of childlessness was 16.8% higher among women with poor CCPKnow scores than among women with adequate or higher CCPKnow scores. Among the respondents, 62.1% (154 of 248) reported having discussed family planning with a physician. Discussion of family planning with a gastroenterologist corresponded with 72% lower odds of voluntary childlessness among childless women
Picciarelli et al. (2022)	United states	To explore the reproductive health decision‐making experiences and preferences of women with IBD	21 females	Qualitative interviews	A researcher developed interview guide was used to explore participants' experiences Nulliparous women who do not currently desire pregnancy appear to lack reproductive health knowledge. Women with IBD lack clarity regarding the role IBD plays in contraceptive choice. Related to pregnancy, women are concerned about the heredity of IBD, antepartum disease activity, and the safety of their current medications. Women with IBD typically default to their reproductive health provider for reproductive health care and counselling, but they expect their gastroenterologist to initiate relevant reproductive health discussions and provide information in the context of their disease
Rao et al. (2021)	United States	To assess counselling and knowledge about IBD and reproductive health	54 females 46 males	Quantitative multiple choice questionnaire	Participants completed the CCPKnow questionnaire. Participants had: UC (52%) and CD (41%), with a small proportion (7%) with indeterminate colitis. Patients reported being counselled on at least one topic (heritability, fertility and IBD, or fertility and medication use) only 33% of the time. Few patients report having been counselled on issues with sexual function and IBD or sexual function and surgery (3% and 15%, respectively) Both men and women considered not having a child due to IBD (31% women, 15% men, 24% total). The majority of people who considered voluntary childlessness were not previously counselled on IBD and reproductive health issues (83%). Many of the patients who became pregnant after their IBD diagnosis did not seek care from a gastroenterologist preconception (38%) and 25% did not seek care from a gastroenterologist during pregnancy. One‐third (33%) stopped or changed their medications during the pregnancy and 40% of these patients did not discuss these medication changes with a physician. The majority of patients (67%) reported an interest in receiving more information on IBD and reproductive health, mostly in the form of a handout (84%) and a clinic visit (69%)
Toomey and Waldron (2013)	Ireland	To assess the knowledge of issues surrounding planning and carrying a pregnancy with IBD	73 female patients 49 GPs	Quantitative prospective questionnaire	Nine patients had CD and 22 had UC. 42% of participants reported that they would allow having IBD to influence their family planning decisions. A total of 77% felt that there was a need to discuss issues with their GP, 32% had such a discussion previously and 58% would like their GP to raise these issues in the future. Only 16% knew that surgery for IBD could potentially lessen their fertility, 68% of patients reported that they are anxious or worried about the effect that their drugs could have on a pregnancy. Most GP's reported that they never provided advice on family planning (57%), planning medication changes before a planned pregnancy (55%) or initiated medication changes during pregnancy (57%). 18% of GP's would routinely raise the issue of family planning with IBD patients when the opportunity arose while 41% said that they never do. 8% of GPs felt they had the expertise to deal with family planning issues if approached by a patient themselves, 31% would have to research it first and 61% would defer to the patient's specialist team for advice 67% of GPs reported that they would defer to the patient's specialist for most decisions about pregnancy
Walldorf et al. (2018)	Germany	To identify patients with an increased need for medical counselling	443 females	Quantitative internet‐based questionnaire	Researcher developed questionnaire Childlessness was reported frequently (64.8% of IBD women). The frequency of childlessness was not significantly different from that in the general population Overall, 13% of women with IBD who were childless avoided pregnancy following their physicians' advice with respect to IBD Because of IBD, family planning was postponed in 57% of IBD mothers. Notably, 38.5% of the mothers with IBD who had postponed family planning did so following medical advice. Overall, 45.7% of the women agreed that IBD had a considerable impact on family planning and pregnancy (FPP) Satisfaction with the physicians' counselling related to IBD in general was higher than the satisfaction with medical advice related to FPP specifically (44.2% vs. 27.3%)

### 3.2 Evaluation of Data

Methodological quality of all empirical studies included in this review was assessed using the Mixed Methods Appraisal Tool (MMAT) (Hong et al. 2018). Authors one and two independently appraised methodological quality. Qualitative and quantitative designs were appraised using five MMAT items, with each item requiring a ‘Yes’, ‘No’, or ‘Can't tell’ response. Discrepancies were discussed in conjunction with the MMAT framework until consensus was reached by all authors. All papers were found to be of good methodological quality (see Table 2), however, a qualitative paper by Picciarelli et al. (2022) did not discuss the qualitative approach applied in the study. Likewise, five quantitative studies did not account for confounders (Calvet et al. 2018; Ellul et al. 2019; Nisihara et al. 2020; Toomey and Waldron 2013; Walldorf et al. 2018), one did not discuss data analysis (Ellul et al. 2019) and one did not discuss the survey instrument used (Toomey and Waldron 2013). As described by Popenoe et al. (2021) an article matrix was developed which assisted with identifying patterns across studies. A biopsychosocial model of health highlights an interconnection between physical, psychological and social influences (Engel 1977; WHO 2002). Three themes which are informed by the authors biopsychosocial understanding of health were developed through an iterative process. These themes are: (1) IBD and sexual functioning; (2) IBD negatively impacts sexual and reproductive health and (3) reproductive knowledge and reservedness to discuss sexual health.

#### 3.2.1 Themes

##### 3.2.1.1 IBD Negatively Impacts Sexual, Reproductive and Social Health

Three papers included in this review considered the broader elements of sexual health which highlight the interplay between physical functioning and an individual's emotional, mental and social wellbeing (Calvet et al. 2018; Knowles et al. 2013; Roseira et al. 2020). Calvet et al. (2018) administered the UC‐LIFE survey. They highlighted there was a higher proportion of Spanish women than men who identified that frequently or mostly/always, relationships with friends and family had been negatively impacted due to UC and that their leisure activities were also negatively influenced by their illness. Of 48 participants, a higher proportion of women (28.4%) compared to men (7.4%) reported that their decision to have children was influenced by their diagnosis of UC. Additionally, of 104 participants more women (52%) compared to men (30%) identified that their diagnosis of UC influenced their ability to care for their children.

Knowles et al. (2013) used a range of scales to explore relationships between sexual health and satisfaction, body image and psychological status. These scales included an illness perception questionnaire, an anxiety and depression scale, sexual problems and sexual satisfaction scales, martial and family functioning scales, and a body image and self‐consciousness during intimacy scale (see Knowles et al. 2013 for a detailed description of the scales). The results of this study highlight the complex relationship between a range of variables and that when a woman perceives poor health due to their IBD this increased their overall anxiety and depression. The study findings highlighted the relationship between heightened psychological distress, and an increase in sexual problems, poor body image and self‐consciousness, lower sexual satisfaction and family functioning. Overall Knowles et al. (2013) found that more Australian women (*n* = 34), compared to Australian men (*n* = 3) with IBD, reported sexual problems and more women identified a lack of sexual interest (*n* = 51) compared to men (*n* = 7), but it was the intersections between the above mentioned variables which added complexity to the individual experience.

Sexual quality of life (SQOL) in IBD along with Social Desirability (SDS‐SF), depression spectrum disorders (PHQ‐9) and health‐related quality of life (HRQoL) was explored by Roseira et al. (2020) highlighted that the limitations of the HRQoL were the failure to consider sexual health more broadly. As also identified by Knowles et al.'s (2013) Australian study, Roseira et al. (2020) found that for the Portuguese participants in their study, there was an interplay between psychological status and self‐esteem and this in turn negatively impacted sexual health. Although there was no statistical significance, women with IBD had lower SQoL scores than women without IBD. Both women and men who lived with IBD had higher levels of frustration, depression, anxiety, embarrassment, guilt, lack of pleasure and loss of self‐confidence (Roseira et al. 2020). While women with IBD in the study reported satisfaction with the frequency of sexual activity, they did report low self‐esteem and avoidance of sexual activity (Roseira et al. 2020). Inclusion of the PHQ‐9 showed that women participants with or without IBD who scored as having moderate to severe depression had lower SQoL.

##### 3.2.1.2 IBD and Sexual Functioning

Sexual functioning following a diagnosis of IBD was explored in several studies included in this review. Most commonly, the validated Female Sexual Functioning Index (FSFI) was used in the studies reviewed (Nisihara et al. 2020; Bel et al. 2015; Boudiaf et al. 2021; Domislovic et al. 2021; Marín et al. 2013; Riviere et al. 2017; Shmidt et al. 2019). It is a 19‐item tool which explores respondents' desire, arousal, lubrication, orgasm, satisfaction and pain. Sexual dysfunction is reported when scores on the FSFI are below 26.55 (Rosen et al. 2000). While the FSFI was used across several studies, the results varied significantly. Shmidt et al. (2019) found in the United States of America, 97% of 116 women participants across all age groups reported sexual dysfunction, which the authors explained was 47% higher than the general population. In the study conducted by Nisihara et al. (2020) in Brazil, 82.5% of the 40 women participants reported sexual dysfunction and a Croatian study found that 75% of the 80 women who completed the FSFI had sexual dysfunction (Domislovic et al. 2021).

Boudiaf et al. (2021) explored sexual dysfunction specifically for those who had a diagnosis of perianal CD and found that of the 64 French women who participated in their study, 66% had sexual dysfunction. Riviere et al. (2017) reported that of the 192 French women with IBD in their study 53.6% had sexual dysfunction scores and similarly, a Spanish study found that almost half of their participants had sexual dysfunction (Marín et al. 2013). These findings were comparable to that of Bel et al. (2015), who found that in the Netherlands, of 168 women included in their study 51.2% had sexual dysfunction. However, in contrast to the finding reported above by Shmidt et al. (2019), there was only a 6.9% difference between women with IBD and the control group of women. Notably Bel et al. (2015) highlighted that the sexual dysfunction scores increased for women by almost 20% when they identified as having active disease compared to women in remission from IBD. More issues with lubrication and painful intercourse were reported by women with active IBD (Bel et al. 2015).

Bulut and Törüner (2019) used the Arizona Sexual Experience Scale (ASEX), which can be administered to both men and women. For women participants, the ASEX explores sexual impulse, arousal, vaginal lubrication and orgasm. High scores on the ASEX indicate sexual dysfunction (Soykan 2002). In the study by Bulut and Törüner (2019), Turkish women with UC and CD had lower ASEX scores than women in the control group. Studies by Bulut and Törüner (2019) using the ASEX and Bel et al. (2015) using the FSFI, found that women with active disease had higher scores, indicating a higher level of sexual dysfunction. Eluri et al. (2018) also used a measure which could be administered to both men and women called the Patient Reported Outcome Measurement Information System (PROMIS) Sexual Function and Satisfaction measure (SexFS). The tool asks participants to reflect on their sexual function and satisfaction from the past 30 days and was designed to be used with men and women with or without chronic conditions (Weinfurt et al. 2015). Lower SexFS scores indicated lower interest and lower satisfaction for women in this study (Eluri et al. 2018). Eluri et al. (2018) highlighted a small but clinically meaningful difference in sexual interest. The authors explained that as depression is more common in patients with IBD this would in turn reduce interest in sexual activity for American women with IBD compared to the general population (Eluri et al. 2018).

Nøhr et al. (2020) adapted questions from the Danish National Health Survey, which specifically explored if the sexual needs for women had been met in the past 12 months. Of the 380,011 participants from Denmark, 605 were women with IBD. The participants were asked about frequency of sexual activity with a partner, pain during intercourse, vaginismus, lubrication, difficulty reaching orgasm and sexual desire. Nøhr et al. (2020) found that painful intercourse was more common for women with CD (17%) compared to women with UC (11%) and women without IBD (10%). Overall there were similar findings between women with CD (67%), UC (66%) and those who did not have IBD (69%) regarding their sexual needs being met and also frequency of sexual activity. Women with CD (19%) reported more difficulty in having an orgasm compared to women with UC (14%) and women without IBD (13%).

##### 3.2.1.3 Limited Reproductive Knowledge and Reservedness to Discuss Sexual Health

Six studies included in this review specifically highlighted that many women who had a diagnosis of IBD had limited knowledge about their reproductive health (Picciarelli et al. 2022; Ellul et al. 2019; Toomey and Waldron 2013; Walldorf et al. 2018; Huang et al. 2015; Rao et al. 2021). Ellul et al. (2019) found that due to lack of knowledge, more than 60% of Mediterranean women who participated in their study were concerned about complications in pregnancy which were either directly related to the disease or complications from the medications. Concerns regarding medication use during pregnancy were also identified by Toomey and Waldron (2013) in Ireland and Walldorf et al. (2018) in Germany, with several women believing that medications should be discontinued during pregnancy. Many participants across studies were anxious about the effect treatments would have on the developing foetus (Ellul et al. 2019; Toomey and Waldron 2013; Walldorf et al. 2018). Huang et al. (2015) found that Canadian women with IBD who were of childbearing age and did not have children had lower Crohn's and Colitis Pregnancy Knowledge scores (CCPKnow). The results, however, did not clearly outline the reasons why women had not had children. While the reason was not clear, the lower reproductive knowledge finding aligned with what Picciarelli et al. (2022) found in the United States of America (USA) through semi‐structured interviews. Picciarelli et al. (2022) highlighted that lower reproductive knowledge could be explained for some women as they were not at a point in their lives where they had considered having children. However, they identified that when it was relevant, they would seek out information. Additionally, Picciarelli et al. (2022) found that women had concerns about the disease flaring during pregnancy and the safety of medications, but also, they expressed concerns regarding heredity of IBD. Rao et al. (2021) highlighted participants in their American study had poor CCPKnow scores, with 24% reporting considering voluntary childlessness due to the same reasons highlighted by Picciarelli et al. (2022).

Interestingly, Rao et al. (2021) highlighted that while participants in their American study had poor reproductive IBD knowledge, they identified that there was a clinical communication gap, with many women not seeking reproductive health counselling. Many of the women in this study had limited contact with physicians, gastroenterologists and obstetricians, and they made decisions to stop medications during pregnancy without consulting clinicians. Furthermore, when reproductive counselling had not been received, this increased the participants consideration of voluntary childlessness (Rao et al. 2021). Additionally, Toomey and Waldron (2013) identified that medications that were considered high risk to the foetus during pregnancy would have been ceased by Irish women who participated in their study. This was despite evidence suggesting that high‐risk medications should be continued, as these reduce the risk of complications during pregnancy compared to active disease.

While reproductive IBD knowledge is reported to be quite low for women who have IBD as identified above, a reservedness to discuss sexual and reproductive health following a diagnosis of IBD was a compounding factor highlighted in the review of the literature. The reservedness was identified in both women who had IBD but importantly also the clinicians (Rao et al. 2021). Rao et al. (2021) identified that 71% of the American women who participated in their study wanted information about their reproductive health however, discussions about fertility were only initiated when women were prescribed methotrexate. Heritability of IBD, fertility and IBD, fertility and medication use, fertility and surgery, sexual function and IBD and sexual function and surgery were discussed with 20% or less of the women participants although all were of reproductive age (Rao et al. 2021).

Both Picciarelli et al. (2022) and Toomey and Waldron (2013) found that most women who participated in their American and Irish studies identified there was a need to discuss IBD and pregnancy; however, a majority of the women would have preferred that the topic be initiated by the GP or their specialist. Toomey and Waldron (2013) however, found that a majority of the Irish GP's (67%) who participated in their study identified that they would refer the women to their specialists for advice regarding family planning. The reason for the Irish GP's deferring to the specialists was that almost half of the GPs who participated in the study (45%) identified that they had never researched the topic of family planning and IBD (Toomey and Waldron 2013). Walldorf et al. (2018) highlighted that German women who participated in their study identified both unsatisfactory and untimely discussions regarding family planning and pregnancy with their physicians, reinforcing a reservedness to talk about sexual health.

## 4 Discussion

There has been a shift from the reductionist view of the17th century philosopher, Rene Descartes which separates the mind and the body to Merleau‐Ponty's philosophy that humans interact with the world through their intellectual bodies (Keller 2020). The International Classification of Functioning, Disability and Health (ICF) is a biopsychosocial model that demonstrates the dynamic, interactive relationship between a person's health condition and a person's contextual factors (World Health Organisation 2002), which aligns with Merleau‐Ponty's philosophy. The way women perceive themselves within their social world has also long been explored within the psychology and social science literature (McLean and LaGuardia 2016; Paquette and Raine 2014). However, how women perceive themselves following a diagnosis of a chronic illness such as IBD is insufficiently researched; what may be profoundly disabling for one person will differ significantly for another given the interplay of physical and contextual factors mentioned above. The complex interrelationship of biological, psychological, socio‐cultural and relationship factors which influence health more broadly is equally influential in sexual and reproductive health for women (Chrisler et al. 2020; Velten and Milani 2020).

The sexual difficulties women experience related to a diagnosis of IBD have been identified both over a short period of time and longitudinally in this review of the literature. Unsurprisingly more severe active IBD was strongly associated with sexual dysfunction for women across studies reviewed (Bel et al. 2015; Shmidt et al. 2019). Although, even when disease activity had settled, Shmidt et al. (2019) found that women with IBD over time continued to experience sexual difficulties highlighting that IBD negatively impacts sexual and reproductive health. Kleiplatz et al. (2020) highlight that many of the diagnoses which are categorised as a “sexual dysfunction” arise from a Western cultural lens. The authors caution that while these appear to be ‘value‐neutral and free from bias’ (Kleiplatz et al. 2020, 443) we must remain open to the critique that cultural norms differ, and the Western model may problematise deviations from what is socially and culturally constructed as normative. The authors recognise that as papers only written in English were included in this review that the western lens may also be perpetuated. The other note of caution is that sexual dysfunctions should not be considered in isolation because they are intrinsically linked to the contextual factors of an individual and the social worlds they inhabit (Kleiplatz et al. 2020). The intrinsic links are highlighted by Bel et al. (2015) as the authors report the interrelationship between active IBD, depressed mood and sexual function. Additionally, higher levels of fatigue, lower disease related quality of life and more negative body image were variables which were also associated with poorer sexual functioning (Bel et al. 2015).

Unfortunately, burdened health care systems, the global attrition of general practitioners and misconceptions regarding the scope of practice for nurses working in general practice are barriers in providing adequate time for complex discussions with people who live with chronic conditions (McInnes et al. 2015). Additionally, as highlighted by Walker and Peterson (2021), health care is rarely universal in the fact that there are many out of pocket expenses such as prescription medications, fees to see GP's, specialist doctors and allied health care professionals which health care systems and health insurances do not cover in their entirety. Furthermore, geographical location can impede access to healthcare as specialist services are not as readily available in rural and remote regions compared to metropolitan areas (Henderson et al. 2018). Such organisational and system constraints can lead to a disease focused model of care which is contrary to the biopsychosocial model of the ICF (World Health Organisation 2002). Unfortunately, this may lead to an inadvertent message that women's sexual and reproductive health is an unimportant aspect of their broader health.

This literature review highlights the multifaceted sexual difficulties women who live with IBD have, such as painful intercourse, vaginismus, lower sexual satisfaction and desire and difficulty with orgasm (Nisihara et al. 2020; Bel et al. 2015; Boudiaf et al. 2021; Domislovic et al. 2021; Marín et al. 2013; Riviere et al. 2017; Shmidt et al. 2019). The literature also highlights that there is a bidirectional relationship between depression and sexual dysfunction (Knowles et al. 2013; Roseira et al. 2020) which has been described in the broader literature by Velten and Milani (2020). An additional intersecting factor which adds complexity is that women who live with chronic conditions (such as IBD), are also more likely to experience sexual difficulties (McCabe et al. 2016). Furthermore, the pervasive socially constructed and gendered prescriptions regarding sexuality profoundly influence the lives of both women and men be it as a consumer or as a clinician who provides support to those in need (Sakaluk et al. 2014). It is thought that the reservedness to discuss sexual and reproductive health highlighted by the women participants in the papers included in this review of the literature is twofold. Firstly, one aspect of this reservedness is thought to be underpinned by sexual scripting which is a gendered organisation of sexuality (Klein et al. 2019; Simon and Gagnon 1986). This pervasive, prescriptive discourse infers that women's sexuality must be suppressed resulting in it being considered a taboo topic which must be veiled in secrecy (Velten and Milani 2020; Sakaluk et al. 2014). While the rhetoric is slowly changing, raising consciousness for clinicians about the diversity of social normative behaviours which influences both men and women is an important first step in discussing sexual and reproductive health as part of everyday healthcare. Secondly, another reason for the reservedness to discuss sexual and reproductive health appears to come from some GP's who did not feel adequately equipped to suitably guide women regarding the management of IBD and family planning (Toomey and Waldron 2013). Toomey and Waldron (2013) highlight that GP's contact with women who have IBD and who are pregnant is rare thus opportunities to develop and maintain contemporary expertise is limited. Kashkooli et al. (2015) found that GP's had the lowest overall specific IBD pregnancy related knowledge compared to obstetricians/gynaecologists and gastroenterologists (whom had the highest knowledge). This highlights that frequency of exposure to pregnant women with IBD is an important factor in ensuring appropriate and safe pregnancy and disease management for women. Kashkooli et al. (2015) suggest that proactive pre‐pregnancy counselling should be initiated by gastroenterologists for all women of childbearing age as this may reduce unnecessary anxiety for not only the women but also for GP's and obstetricians/gynaecologists who may be seeing women after they conceive. This aligns with what some women in the studies included in the literature review indicated. Women wanted to talk about their sexual and reproductive health because they did have concerns about the impact of IBD on pregnancy but rather than self‐initiate these discussions they wanted this to be instigated by the health care provider they were seeing (Picciarelli et al. 2022). Hansen et al. (2022) found women requiring maternity services who lived with a range of chronic medical conditions felt as though they were monitored although they did not feel sufficiently supported to navigate the healthcare system. Access to specialist services and clear referral pathways are critical elements in ensuring women have access to evidence‐based information which can guide their decision making. However Lui et al. (2023) highlight that there is a need for ongoing education to ensure high quality standardised care for women with IBD who are pregnant.

Active IBD during pregnancy can lead to adverse birth outcomes and the clinical recommendation is one of remission before considering pregnancy (Bröms et al. 2021). The literature highlights that there is a greater need for family planning and reproductive health knowledge for women who live with IBD. Many women across studies included in this review identified concerns regarding the effect medications would have on a developing foetus (Ellul et al. 2019; Toomey and Waldron 2013; Walldorf et al. 2018). This concern by women is not unfounded as Meyer et al. (2021) found that thiopurines, which are used in the treatment of IBD to reduce the activity of the immune system, more frequently resulted in adverse outcomes such as stillbirths and pre‐term births and larger babies. The authors of this paper do note in the nationwide study conducted in France that the reduction in the use of thiopurines during the 8 years of data collection coincided with an increasing use of TNF inhibitors. The use of TNF inhibitors, which are used to stop inflammation, was not associated with the same adverse birth outcomes (Meyer et al. 2021).

Discussions regarding reproduction were the most prevalent within the literature. While reproduction is an important aspect of women's lives, this finding highlights that sexual health should not be limited to reproduction and sexual dysfunction but should be more holistically focused on women's sexual wellbeing (Fourie et al. 2021). Due to the chronic nature of IBD and its presence across the lifespan, it is imperative that sexuality for women, regardless of their life stage, is considered with a broader lens. This broader lens is needed to challenge what Interligi and McHigh (2020) describe as a historical heteronormative and androcentric understanding of what sexuality is. The authors highlight that nurses and midwives are well positioned to ensure conversations regarding sexual wellbeing are part of a continuum of care that includes a variety of settings such as general practice, acute care and maternity services.

## 5 Conclusion

This review of the literature highlights that discussions regarding sexual and reproductive health issues are not consistently included in the management and treatment for women who live with IBD. These discussions remain focused on sexual dysfunction and reproduction. This review brings to light that there is a dearth of information which explores women's sexuality more broadly which includes but is not limited to women's self‐esteem and feelings of personal attractiveness. It identifies that sexual and reproductive health discourse needs to move beyond sexual activity. Additionally, research which is led by nurses and midwives exploring sexual wellbeing for women who live with IBD and other chronic health conditions is required.

## Author Contributions

**Kate O'Reilly:** conceptualisation (equal), methodology (equal), funding acquisition (equal), writing original draft (lead), writing – review and editing (lead). **Susan McInnes:** methodology (equal), writing – review and editing (supporting). **Eleanor Holroyd:** conceptualisation (equal), methodology (equal), writing – review and editing (supporting). **Kath Peters:** conceptualisation (equal), funding acquisition (equal), writing – review and editing (supporting).

## Ethics Statement

The authors have nothing to report.

## Conflicts of Interest

The authors declare no conflicts of interest.

## Supporting information


Data S1.

## Data Availability

The authors have nothing to report.
